# Does the Disparity Patterning Differ between Diagnosed and Undiagnosed Hypertension among Adults? Evidence from Indonesia

**DOI:** 10.3390/healthcare11060816

**Published:** 2023-03-10

**Authors:** Puput Oktamianti, Dian Kusuma, Vilda Amir, Dwi Hapsari Tjandrarini, Astridya Paramita

**Affiliations:** 1 Health Administration and Policy Department, Faculty of Public Health, Universitas Indonesia, Depok 16424, Indonesia; 2 Department of Health Services Research and Management, School of Health & Psychological Sciences, City University of London, London EC1V 0HB, UK; 3 Center for Health Administration and Policy Studies, Faculty of Public Health, Universitas Indonesia, Depok 16424, Indonesia; 4 Research Center for Public Health and Nutrition, National Research and Innovation Agency, Bogor 16915, Indonesia

**Keywords:** diagnosed, undiagnosed, high blood pressure, inequality, geographic, socioeconomic

## Abstract

Background: Healthcare systems in many low- and middle-income countries (LMICs) are not yet designed to tackle the high and increasing burden of non-communicable diseases (NCDs), including hypertension. As a result, a large proportion of people with disease or risk factors are undiagnosed. Policymakers need to understand the disparity better to act. However, previous analyses on the disparity in undiagnosed hypertension, especially from LMICs, are lacking. Our study assessed the geographic and socioeconomic disparity in undiagnosed hypertension and compared it with diagnosed hypertension. Methods: We used the Basic Health Survey (Riskesdas) 2018 and performed geospatial and quantitative analyses across 514 districts in Indonesia. Dependent variables included diagnosed and undiagnosed hypertension among adults (18+ years) and by gender. Results: A high prevalence of undiagnosed hypertension at 76.3% was found, with different patterns of disparity observed between diagnosed and undiagnosed hypertension. Diagnosed hypertension was 1.87 times higher in females compared with males, while undiagnosed hypertension rates were similar between genders. Urban areas had up to 22.6% higher rates of diagnosed hypertension, while undiagnosed hypertension was 11.4% more prevalent among females in rural areas. Districts with higher education rates had up to 25% higher diagnosed hypertension rates, while districts with lower education rates had 6% higher rates of undiagnosed hypertension among females. The most developed regions had up to 76% and 40% higher prevalence of both diagnosed and undiagnosed hypertension compared with the least developed regions. Conclusion: The disparity patterning differs between diagnosed and undiagnosed hypertension among adults in Indonesia. This highlights the need for effective measures, including healthcare system reforms to tackle NCDs in LMICs.

## 1. Background

Hypertension, or high blood pressure, is linked with increased heart, brain, and kidney disease risks [1]. Globally, about 1.3 billion adults aged 30 years and over had hypertension in 2021. Most of those with hypertension (over 60%) are in low- and middle-income countries (LMICs) [1]. Moreover, data also showed that less than half (42%) of those with hypertension were diagnosed and treated [1]. In Indonesia, hypertension is also high and increasing. Analyses from the nationally representative survey (RISKESDAS) showed that hypertension prevalence among adults 18 years and over was 34.1% in 2018, which increased considerably from 25.8% in 2013 [2]. Moreover, a study of the Indonesian Family Life Survey 2016 found that the prevalence of hypertension among adults 40 years and over was 47.8%, of which almost 70% were undiagnosed [3].

The current literature provides some evidence of social determinants of cardiovascular diseases and risk factors including hypertension [4]. A comprehensive literature review and meta-analysis in 2014 found that income level was positively associated with hypertension, but education level was not. The study also found geographic variation in the association between education and hypertension, showing an inverse association in the East Asian region and a positive one in the South Asian region [5]. A study in 2017 using the South Korean National Health and Nutrition Examination Survey (NHNES) found sexual variation in the association between education and undiagnosed hypertension, showing an inverse association among women but not among men [6]. Recent analyses (2019–2021) of the Demographic & Health Survey data in Peru, Bangladesh, and Nepal also found that adults from lower socioeconomic and educational backgrounds had higher odds of undiagnosed hypertension [7,8,9,10]. Another study in 2016 showed that being in a deprived neighborhood increased the influence of individual socioeconomic status on mortality among newly diagnosed hypertension patients in South Korea [11]. Similarly, a study in Peru found that adult males living in the more remote and deprived areas (e.g., coasts and mountains) had a higher prevalence of undiagnosed hypertension [7]. A study in the United States showed that rural areas were most vulnerable to adverse chronic health outcomes and found a positive association between social vulnerability index and cardiometabolic indicators including hypertension [12].

To achieve the SDG target 3.4.1 to reduce premature mortality from NCDs by one-third by 2030, efforts need to aim at reducing the disparity in diagnosed and undiagnosed hypertension [1]. However, the current literature on such disparity is limited in three ways. First, while most of the current literature used data at the individual level (e.g., national surveys) [3,5,6,7,8,9,10], studies that employed data at the local level (such as districts) are lacking. Such evidence is also crucial, especially in countries with more local decision space, such as Indonesia. Second, because of the better availability of local level data, current geographic analyses are mainly from high-income countries such as the United States and South Korea [11,13,14]. Such analyses from LMICs (e.g., China, Thailand, and Peru) are limited to the urban/rural and provincial levels [15,16,17]. Third, previous studies focused on overall hypertension and lacked disaggregation between diagnosed and undiagnosed hypertension. Effective health system reforms and population-based interventions may be needed to reduce the undiagnosed population [18]. Our study aimed to assess the disparity (geographic and socioeconomic) in diagnosed and undiagnosed adult hypertension across over 500 Indonesian districts.

## 2. Methods

### 2.1. Study Design

This is a cross-sectional study comparing the disparity in diagnosed and undiagnosed hypertension among adults. We analyzed geographic and socioeconomic disparities across 514 districts within 34 provinces in Indonesia. We took advantage of the 2018 Basic Health Survey (Riskesdas) data that were representative at the district level for diagnosed and undiagnosed hypertension. The survey conducted interviews and physical examinations of about 300,000 households from a two-stage sampling procedure. The sampling first randomly selected 30,000 census blocks (out of a total of over 700,000 in Indonesia). Within each block, 10 households were systematically selected, which resulted in 624.563 adults (18+ years). The mean ages (standard deviation) were 41.0 (15.5) years, 40.8 (15.3) years, and 41.3 (15.7) years for all adults, males, and females, respectively [2].

### 2.2. Independent Variables

The main independent variables included geographic and socioeconomic indicators at the district level. The variables used in our analyses were region, urbanicity, income level, and education level. This information was taken from the World Bank. The regional variable includes five regions: Sumatera, Java (including Bali), Kalimantan, Sulawesi, and Papua (including Nusa Tenggara and Maluku). Generally, the eastern parts of the country are the least developed [19,20,21]. Appendix A provides the map reference. The urbanicity variable shows cities as urban and regencies as rural areas. For the income variable, we used the poverty rates information at the district level, which we then grouped into quintiles. For the education variable, we used net enrollment ratios of senior secondary information, which we grouped into quintiles as well [22,23,24].

### 2.3. Dependent Variables

There were six dependent variables used in our analysis, including diagnosed adults, diagnosed males, diagnosed females, undiagnosed adults, undiagnosed males, and undiagnosed females. Diagnosed hypertension was a binary variable with a value of 1 if one reported ever being told by a doctor that they have high blood pressure and 0 if otherwise. We defined undiagnosed hypertension as not diagnosed but meeting the criteria for hypertension based on the blood pressure measurement (i.e., either systolic blood pressure of at least 140 mmHg, diastolic blood pressure of at least 90 mmHg, or both) [25].

### 2.4. Data Analysis

We performed both geospatial analyses and multivariable regression analyses in this paper. In conducting the geospatial analyses, we grouped each dependent variable for 34 provinces and 514 districts by quintile. In conducting the regressions, we employed ordinary least squares and examined the relationship between independent and dependent variables. We compared the regional variations between the western and eastern parts of the country, and the income/education variations between the poorest/least educated and wealthiest/most educated. The geospatial analyses were conducted in ArcMap 10 and the statistical analyses were performed in STATA 15, using 5% as statistically significant.

## 3. Results

### 3.1. Analysis at the Provincial Level

**Figure 1:**
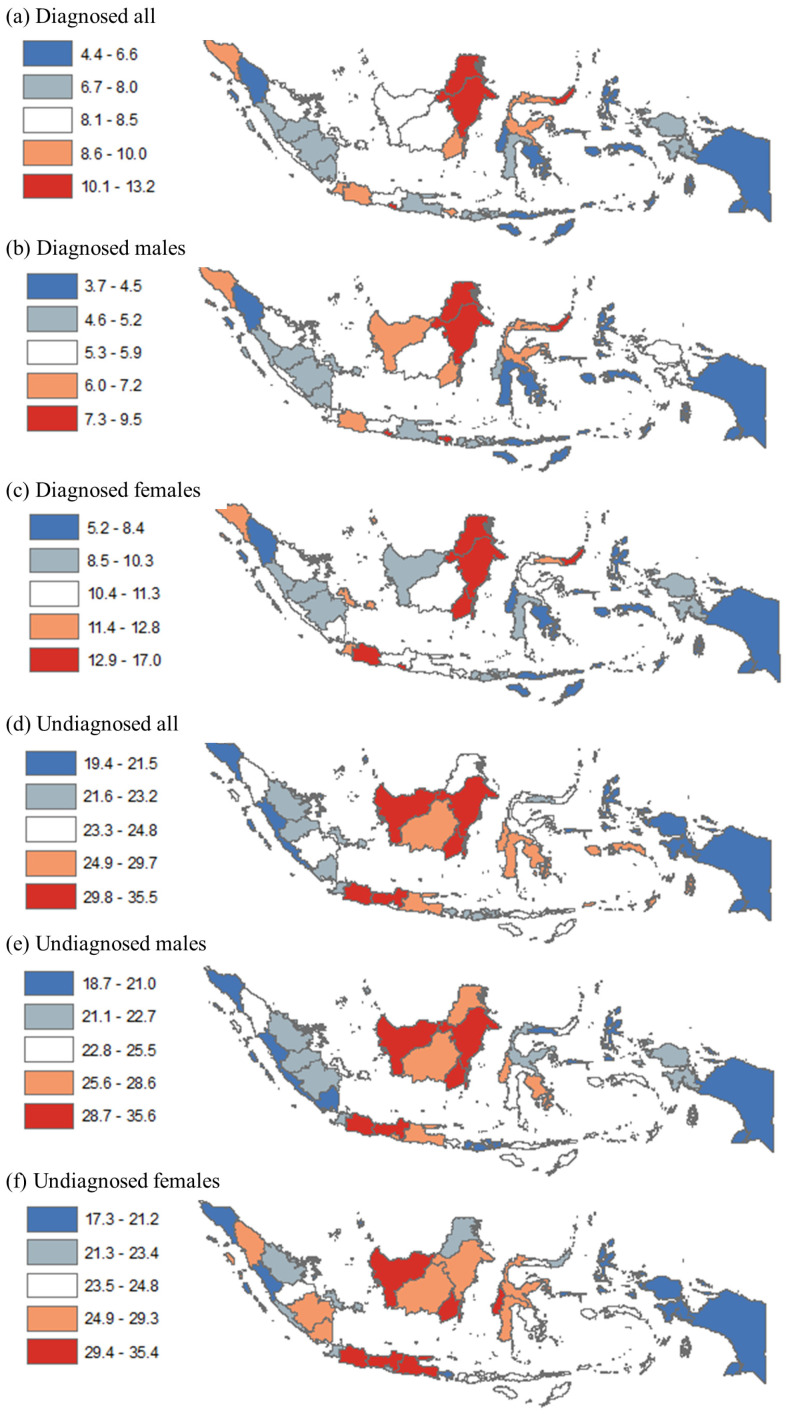
Disparity in diagnosed and undiagnosed hypertension among adults (18+ years) by province in Indonesia, 2018. Note: Numbers show prevalence of diagnosed and undiagnosed hypertension among all adults, males, and females.

Figure 1 and Table 1 show results at the provincial level. Figure 1 compares diagnosed hypertension (panels a–c) and undiagnosed hypertension (panels d–f) by quintile. At the provincial level, diagnosed hypertension among all adults ranged from 4.4% to 13.2%; males from 3.7% to 9.5%; and females from 5.2% to 17.0%. At that level, undiagnosed hypertension among all adults ranged from 19.4% to 35.5%; males from 18.7% to 35.6%; and females from 17.3% to 35.4%. Diagnosed hypertension among all adults was highest (quintiles four–five) in many provinces in the Java and Bali region (e.g., Jakarta, Banten, West Java, Yogyakarta, and Bali), several provinces in Kalimantan (e.g., East, North, and South Kalimantan) and Sulawesi (e.g., North Sulawesi, Central Sulawesi, and Gorontalo), and a province in Sumatera (i.e., Aceh). Undiagnosed hypertension among all adults was highest (quintiles four–five) in many provinces in Java (e.g., Jakarta, West Java, Central Java, East Java, and Bali) and Kalimantan (e.g., East, West, Central, and South Kalimantan), many provinces in Sulawesi (e.g., West, South, and Southeast Sulawesi), and two provinces in Sumatera and Papua. By sex, the patterning showed some differences. For instance, diagnosed hypertension among females was higher (quintile four) and that among males was lower (quintile two) in Bangka Belitung. In contrast, diagnosed hypertension among females was lower and that among males was higher in West Kalimantan. Similarly, undiagnosed hypertension among females was higher, and that among males was lower in North Sumatera, South Sumatera, and Lampung.

Moreover, Table 1 compares diagnosed hypertension and undiagnosed hypertension by the level of poverty rates at the provincial level. The top box and bottom box compare the ten richest and poorest provinces. The provincial prevalence higher than the national level is shown in grey in each column. Of the ten wealthiest provinces, six provinces (e.g., Jakarta, Bali, South, North, and East Kalimantan) had higher prevalence than average for at least four indicators, while none of the ten poorest provinces did.

### 3.2. Analysis at the District Level

**Figure 2:**
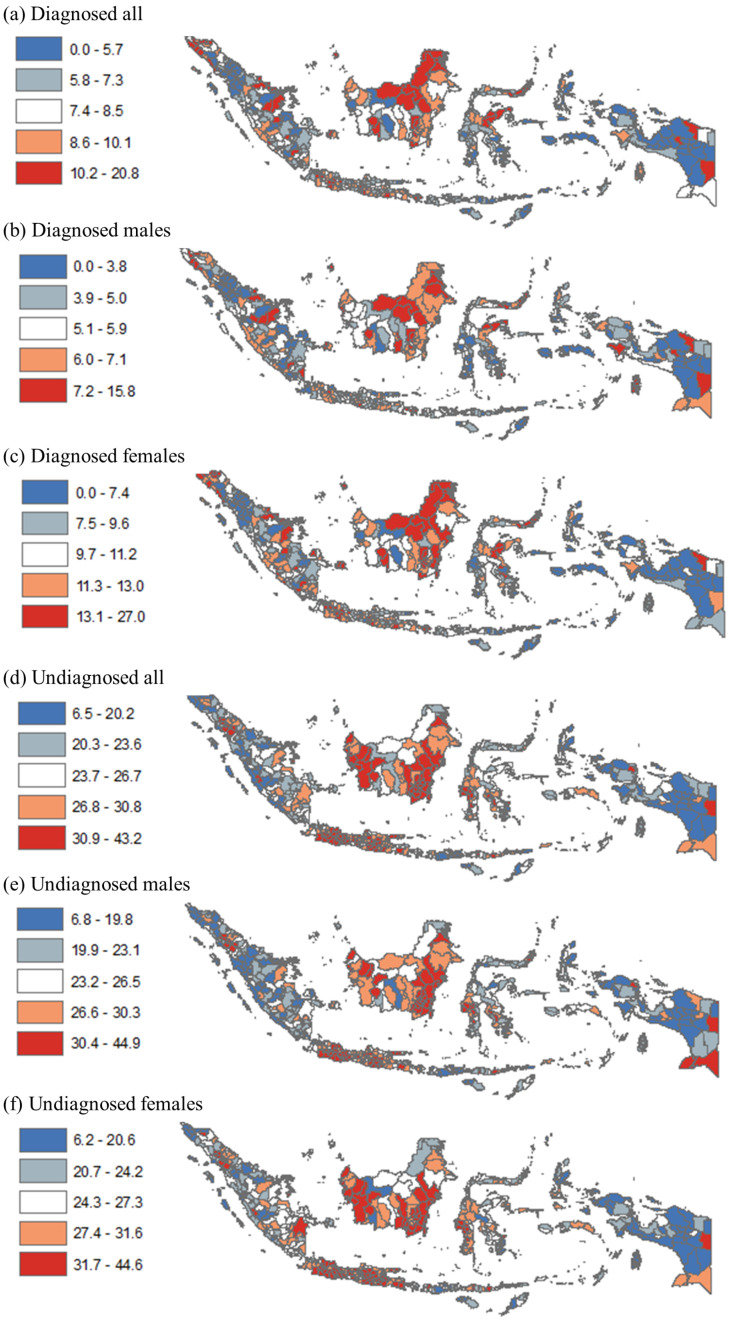
Disparity in diagnosed and undiagnosed hypertension among adults (18+ years) by district in Indonesia, 2018. Note: Numbers show prevalence of diagnosed and undiagnosed hypertension among all adults, males, and females.

Figure 2 and Table 2 and Table 3 show results at the district level. Table 2 shows the characteristics of districts in terms of geographic indicators, socioeconomic indicators, and dependent variables (i.e., diagnosed and undiagnosed hypertension). Of the total of 514 districts in our analysis, 97 (18.9%) and 417 (81.1%) were urban (cities) and rural (regencies). The two regions where urban districts were dominant included Java (36.1% of 97) and Sumatera (34.0%). For the income variable, most of the urban areas (78.4%) were considered wealthy (quintiles four–five), but fewer than a third of rural areas (31.2%) were. Similarly, for education, 71.1% of urban areas had higher education (quintiles four–five), while only a third (32.6%) of rural areas did. In terms of hypertension, diagnosed prevalence was 7.9%, 5.5%, and 10.3%, while that of undiagnosed hypertension was 25.4%, 24.9%, and 25.8% among adults, males, and females. Relative to rural areas, diagnosed hypertension was significantly higher, but undiagnosed hypertension among females was significantly lower in urban areas. Diagnosed hypertension among adults, males, and females was 8.9%, 6.5%, and 11.2% in urban areas and 7.6%, 5.3%, and 10.1% in rural areas. Undiagnosed hypertension among females was 23.4% and 26.4% in urban and rural areas, respectively.

Figure 2 compares the prevalence of diagnosed and undiagnosed hypertension by quintile at the district level. For diagnosed hypertension, many districts in the provinces of Jambi, Riau, Bengkulu, Central Java, East Java, West Kalimantan, Central Kalimantan, South Sulawesi, Papua, and West Papua had higher hypertension among all adults (quintiles four–five). For undiagnosed hypertension, many districts in all provinces in Sumatera and Papua had higher prevalence among adults (quintiles four–five).

For socioeconomic disparity analysis at the district level, Appendix C and Appendix D compare districts with the lowest and highest diagnosed and undiagnosed hypertension. For diagnosed hypertension, the prevalence among adults ranged from 0% in Buton Tengah regency (Central Sulawesi province) to 20.8% in Sitaro Kepulauan (North Sulawesi). By sex, diagnosed hypertension among males ranged from 0% in Yahukimo and Pegunungan Bintang (Papua) to 15.8% in Tomohon city (North Sulawesi); that among females ranged from 0% in Buton Tengah (Southeast Sulawesi), Yahukimo, Dogiyai, and Mambramo Raya (Papua) to 27.0% in Sitaro Kepulauan (North Sulawesi). For undiagnosed hypertension, the prevalence among adults ranged from 7% in Puncak Jaya (Papua) to 43.2% in Hulu Sungai Tengah (South Kalimantan). By sex, undiagnosed hypertension among males ranged from 6.8% in Puncak Jaya (Papua) to 44.9% in Madiun city (East Java); that among females ranged from 6.2% in Puncak Jaya (Papua) to 44.6% in Ciamis (West Java). By urbanicity, districts with the lowest prevalence of diagnosed and undiagnosed hypertension for all adults, males, and females were rural. Similarly, most districts with the highest prevalence of diagnosed and undiagnosed were rural. By income, poverty rates among ten districts with the highest diagnosed and undiagnosed hypertension were averaged up to 10%, while those with the lowest prevalence were averaged up to 33%.

Table 3 compares the associations between geographic/socioeconomic variables and diagnosed/undiagnosed hypertension. Districts in the least disadvantaged region had a significantly higher prevalence of both diagnosed and undiagnosed among all adults, males, and females, relative to the most disadvantaged region (e.g., Papua). Compared with Papua, districts in the Java region had 68%, 45%, and 76% higher diagnosed prevalence among adults, males, and females; they had 40%, 39%, and 40% higher undiagnosed prevalence (significant at 5% level). Moreover, results showed that districts in the Kalimantan region had the highest diagnosed and undiagnosed prevalence among all adults, males, and females in the country. For the income variable, results show that the richest districts had a higher diagnosed and undiagnosed prevalence among all adults, males, and females than the poorest ones (but not statistically significant in multivariable regressions). For the education variable, the relationships are mixed. Districts with the most education had 23%, 18%, and 25% significantly higher diagnosed prevalence among adults, males, and females than the least educated ones. However, districts with the least education had a 6% (i.e., 1/0.94 = 1.06) higher undiagnosed prevalence among females.

## 4. Discussion

Using nationally representative survey data of adults, we found the prevalence of overall hypertension was 33.3%, of which 76.3% were undiagnosed (i.e., 7.9% diagnosed and 10.3% undiagnosed). Global estimates showed similar hypertension prevalence in adults 30–79 years of age at 32% and 34% among women and men in 2019 [26]. In terms of undiagnosed hypertension, while considerably higher than in high-income countries such as the United States (19.7% in 2010), South Korea (33.4% in 2013), and Ireland (41.2% in 2011) [6,27], the prevalence in Indonesia was relatively similar to that in LMICs such as Nepal (50.4% in 2016), Bangladesh (59.9% in 2011 and 50.1% in 2017), and Peru (67.2% in 2019) [7,9,10].

By sex, diagnosed hypertension among females was 1.87 times higher compared with males (i.e., 5.5% males and 10.3% females), while undiagnosed hypertension was similar between both sexes (i.e., 24.9% and 25.8% among males and females). This finding aligns with evidence from other LMICs, such as Nepal, Bangladesh, and Peru, showing a significantly lower prevalence of undiagnosed hypertension among women [7,9,10]. This might be due to women having more interactions with the health systems (e.g., through antenatal, delivery, and postnatal care) and other population-based interventions more towards women (e.g., conditional cash transfers) [28,29].

We found significant disparities (geographic and socioeconomic) between the prevalence of diagnosed and undiagnosed hypertension across 514 districts. Diagnosed hypertension was higher by up to 22.6% in urban areas, while undiagnosed hypertension among females was higher by 11.4% in rural areas. Previous studies showed a higher prevalence of diagnosed hypertension in urban areas but a higher prevalence of undiagnosed hypertension in rural areas [7,8,9,10]. This is expected, as urban areas tend to have higher access to health facilities and healthcare professionals. However, many rural districts were among the top ten districts with the highest prevalence of diagnosed and undiagnosed hypertension, which may be due to similarities in economic development and mobility between rural and urban areas [30]. For example, the North Sulawesi, Minahasa and Minahasa Selatan regencies, which had similar income levels and were adjacent to Tomohon City, were found to have high rates of hypertension.

By region, the patterning is similar for diagnosed and undiagnosed hypertension. Districts in the most developed areas (i.e., Java and Bali) had up to a 76% and 40% higher prevalence of diagnosed and undiagnosed hypertension compared with the least developed areas (i.e., Papua, Nusa Tenggara, and Maluku). This is likely due to a higher burden of hypertension (diagnosed and undiagnosed) among higher socioeconomic levels of the population in more developed regions. By income, the richest districts had a higher prevalence of diagnosed and undiagnosed hypertension among all adults, males, and females than that of the poorest districts (although only statistically significant in bivariate analyses). By education, districts with the most education had up to a 25% higher prevalence of diagnosed hypertension, while those with the least had a 6% higher undiagnosed prevalence among females.

While evidence from LMICs are limited in the literature, our findings align with previous studies. Studies using provincial-level data in China showed a higher prevalence of hypertension in the least disadvantaged areas than that in the most disadvantaged ones [15,16]. Similar study at the provincial level in Thailand found a higher prevalence of hypertension in Bangkok and metropolitan areas than in less developed areas in the north and south regions [17]. On the contrary, studies from high-income countries found a higher prevalence of hypertension in the most disadvantaged areas [11,13,14]. Moreover, a higher prevalence of diagnosed hypertension among districts with the most education may be due to better health systems and access to health facilities [31]. In contrast, analyses at the individual level in Peru, Bangladesh, and Nepal found adults with low education had higher odds of undiagnosed hypertension [7,8,9,10]. Studies have also shown strong association between low education and cardiometabolic comorbidities and that education may be considered the best predictor of cardiovascular risk in people with hypertension [32,33].

Effective efforts are needed to reduce undiagnosed hypertension (and other NCD risk factors such as high cholesterol and diabetes) by sex, urbanicity, region, and socioeconomic status [34,35]. Efforts may include health system reforms such as enhanced primary health care in Malaysia or routine assessment national programs such as NHS Health Check in the United Kingdom [18,36]. Healthcare delivery reforms may also include integration with infectious disease platforms [37,38].

Our study is the first analysis from LMICs to compare the disparity (geographic and socioeconomic) in the prevalence of diagnosed and undiagnosed hypertension across over 500 localities. However, our study also has limitations. Because of the lack of information, our analysis could not conduct sub-group analysis by ethnicity [39]. Additionally, because of using cross-sectional data, our analysis could not conduct trend analysis. However, regardless of these limitations, our evidence is crucial for policymaking nationally and globally, especially in low-resource settings.

## 5. Conclusions

In Indonesia, a high prevalence of undiagnosed hypertension at 76.3% was found with different patterns of disparity observed between diagnosed and undiagnosed hypertension. Diagnosed hypertension was 1.87 times higher in females compared with males, while undiagnosed hypertension rates were similar between genders. Urban areas had up to 22.6% higher rates of diagnosed hypertension, while undiagnosed hypertension was 11.4% more prevalent among females in rural areas. Districts with higher education rates had 25% higher diagnosed hypertension rates, while districts with lower education rates had 6% higher rates of undiagnosed hypertension among females. The most developed regions had up to a 76% and 40% higher prevalence of both diagnosed and undiagnosed hypertension compared with the least developed regions. This study highlights the need for effective measures, including healthcare system reforms, to tackle NCDs in LMICs.

## Figures and Tables

**Table 1:** Prevalence of diagnosed and undiagnosed hypertension among adults (18+ years) by province in Indonesia, 2018.

	Poverty	Hypertension Prevalence (%)					
	Rates	Diagnosed			Undiagnosed		
	(%)	All	Males	Females	All	Males	Females
	[1]	[2]	[3]	[4]	[5]	[6]	[7]
Bali	4.5	9.5	7.8	11.2	22.4	25.0	19.9
South Kalimantan	4.8	10.0	6.8	13.2	35.5	35.6	35.4
Central Kalimantan	5.0	8.4	5.8	11.3	27.6	26.9	28.4
Jakarta	5.0	10.1	7.7	12.5	25.3	27.1	23.5
Banten	5.3	8.6	5.9	11.4	22.8	22.4	23.2
Bangka Belitung	5.4	8.3	4.7	12.3	23.2	23.0	23.4
West Sumatera	6.6	7.2	5.2	9.2	19.8	18.7	20.9
North Kalimantan	7.0	10.5	7.3	14.1	24.8	26.3	23.0
East Kalimantan	7.1	10.6	8.2	13.2	30.6	31.8	29.3
Riau Islands	7.6	8.5	5.8	11.5	19.5	21.7	17.3
Jambi	7.8	7.4	5.1	9.8	22.7	21.5	23.9
North Maluku	7.9	5.7	4.0	7.5	20.7	20.3	21.2
West Java	7.9	9.7	6.3	13.1	31.2	30.5	32.0
West Kalimantan	8.1	8.1	6.1	10.3	30.2	30.0	30.4
North Sulawesi	8.5	13.2	9.5	17.0	23.7	25.5	21.7
Riau	8.8	8.4	5.9	11.0	22.6	21.9	23.4
South Sulawesi	9.8	7.1	4.5	9.4	26.1	24.9	27.3
West Sulawesi	10.3	6.6	5.1	8.1	29.7	28.6	30.7
East Java	10.9	8.0	5.2	10.6	29.7	28.6	30.7
Central Java	10.9	8.1	5.6	10.5	30.6	30.1	31.1
North Sumatera	11.3	5.5	3.8	7.1	24.8	24.7	25.0
Lampung	12.6	8.0	5.1	10.9	23.2	21.0	25.4
Yogyakarta	12.7	10.7	7.3	14.0	24.5	26.8	22.3
Southeast Sulawesi	13.0	6.2	3.9	8.4	24.9	25.6	24.2
South Sumatera	13.1	7.3	5.2	9.5	24.4	22.7	26.2
Central Sulawesi	14.6	8.7	6.3	11.2	23.5	22.2	24.9
West Nusa Tenggara	14.8	7.2	5.2	9.0	22.1	19.3	24.6
Bengkulu	15.0	8.4	5.5	11.3	21.5	20.4	22.6
Aceh	16.4	9.4	6.3	12.3	19.5	18.9	20.0
Gorontalo	16.8	10.0	7.2	12.8	22.7	21.0	24.4
Maluku	21.8	5.0	4.0	5.9	25.0	25.2	24.8
East Nusa Tenggara	22.0	5.4	4.0	6.7	23.6	23.3	23.9
West Papua	26.5	7.4	5.6	9.4	20.6	22.2	18.9
Papua	29.4	4.4	3.7	5.2	19.4	20.3	18.4
Average		8.2	5.8	10.6	24.7	24.5	24.8

Note: Ordered by the average poverty rates (column 1), the provinces in the top box are richest and those in the bottom box are poorest. Shaded values show higher than the national average prevalence for each group.

**Table 2:** Characteristics of districts and hypertension (diagnosed and undiagnosed) among adults (18+ years) in Indonesia, 2018.

		All		Urban		Rural		Difference
		n	%	n	%	n	%	%
		[1]	[2]	[3]	[4]	[5]	[6]	[7] = [4,5,6]
(a) Characteristics (#)								
Sample size district		514	100%	97	100%	417	100%	0%
Region								
	Papua	95	18.5%	9	9.3%	86	20.6%	11.3%
	Java	128	24.9%	35	36.1%	93	22.3%	−13.8%
	Sumatera	154	30.0%	33	34.0%	121	29.0%	−5.0%
	Kalimantan	56	10.9%	9	9.3%	47	11.3%	2.0%
	Sulawesi	81	15.8%	11	11.3%	70	16.8%	5.4%
		514		97		417		
Income/poverty								
	Q1 poor	102	19.8%	3	3.1%	99	23.7%	20.6%
	Q2	103	20.0%	5	5.2%	98	23.5%	18.3%
	Q3	103	20.0%	13	13.4%	90	21.6%	8.2%
	Q4	103	20.0%	22	22.7%	81	19.4%	−3.3%
	Q5 rich	103	20.0%	54	55.7%	49	11.8%	−43.9%
		514		97		417		
Education								
	Q1 least	103	20.0%	0	0.0%	103	24.7%	24.7%
	Q2	103	20.0%	11	11.3%	92	22.1%	10.7%
	Q3	103	20.0%	17	17.5%	86	20.6%	3.1%
	Q4	103	20.0%	29	29.9%	74	17.7%	−12.2%
	Q5 most	102	19.8%	40	41.2%	62	14.9%	−26.4%
		514		97		417		
(b) Hypertension (%)								
Diagnosed all		n/a	7.9%	n/a	8.9%	n/a	7.6%	1.3% *
Diagnosed males		n/a	5.5%	n/a	6.5%	n/a	5.3%	1.2% *
Diagnosed females		n/a	10.3%	n/a	11.2%	n/a	10.1%	1.1% *
Undiagnosed all		n/a	25.4%	n/a	24.7%	n/a	25.5%	−0.8%
Undiagnosed males		n/a	24.9%	n/a	26.1%	n/a	24.7%	1.4%
Undiagnosed females		n/a	25.8%	n/a	23.4%	n/a	26.4%	−3.0% *

Note: Q = quintile, n = number, % = proportion of column total, Urban = city, Rural = regency. Data on district characteristics are from the World Bank and hypertension data are from Basic Health Survey 2018. Bold numbers with asterisk (*) show statistical significance at 5% level (see Appendix B for the regression outputs).

**Table 3:** Geographic and socioeconomic disparity in diagnosed and undiagnosed hypertension among adults (18+ years) in Indonesia, 2018.

		All Districts (n = 514)						Urban (n = 97)						Rural (n = 417)					
		Diagnosed			Undiagnosed			Diagnosed			Undiagnosed			Diagnosed			Undiagnosed		
		All	Males	Females	All	Males	Females	All	Males	Females	All	Males	Females	All	Males	Females	All	Males	Females
		[1]	[2]	[3]	[4]	[5]	[6]	[7]	[8]	[9]	[10]	[11]	[12]	[13]	[14]	[15]	[16]	[17]	[18]
Region																			
	Papua	5.3%	4.2%	6.6%	21.0%	20.9%	21.1%	6.6%	5.0%	8.2%	21.7%	23.2%	20.2%	5.2%	4.1%	6.4%	20.9%	20.6%	21.2%
	Sulawesi	8.4%	5.8%	11.0%	25.6%	25.2%	26.0%	9.9%	7.7%	12.0%	23.4%	25.4%	21.4%	8.1%	5.4%	10.8%	25.9%	25.1%	26.7%
	Kalimantan	9.5%	6.8%	12.3%	30.6%	30.6%	30.5%	10.1%	7.8%	12.5%	28.3%	30.6%	25.9%	9.3%	6.7%	12.3%	31.0%	30.6%	31.4%
	Sumatera	7.7%	5.2%	10.3%	22.8%	21.9%	23.8%	8.0%	5.8%	10.2%	21.9%	22.7%	21.2%	7.7%	5.1%	10.3%	23.1%	21.7%	24.5%
	Java	8.9%	6.1%	11.6%	29.3%	29.0%	29.6%	9.6%	7.0%	12.2%	27.6%	29.1%	26.3%	8.6%	5.8%	11.3%	29.9%	29.0%	30.8%
	Absolute	**3.6%**	**1.9%**	**5.0%**	**8.3%**	**8.1%**	**8.5%**	**3.0%**	**2.0%**	**4.0%**	**5.9%**	**5.9%**	**6.1%**	**3.4%**	**1.7%**	**4.9%**	**9.0%**	**8.4%**	**9.6%**
	Relative	**1.68**	**1.45**	**1.76**	**1.40**	**1.39**	**1.40**	**1.45**	**1.40**	**1.49**	**1.27**	**1.25**	**1.30**	**1.65**	**1.41**	**1.77**	**1.43**	**1.41**	**1.45**
Income																			
	Q1 poor	6.2%	4.7%	7.9%	21.7%	21.3%	22.1%	7.2%	5.8%	8.5%	23.8%	24.4%	23.3%	6.2%	4.7%	7.9%	21.6%	21.2%	22.1%
	Q2	7.3%	4.9%	9.8%	25.3%	24.4%	26.2%	9.0%	6.5%	11.4%	22.9%	23.6%	22.2%	7.3%	4.9%	9.7%	25.4%	24.4%	26.4%
	Q3	8.1%	5.5%	10.7%	27.6%	26.6%	28.5%	8.5%	6.2%	10.9%	23.3%	24.2%	22.5%	8.1%	5.4%	10.7%	28.2%	26.9%	29.4%
	Q4	8.6%	5.8%	11.4%	25.9%	25.4%	26.4%	8.5%	6.1%	10.9%	24.4%	25.2%	23.6%	8.6%	5.7%	11.5%	26.3%	25.4%	27.2%
	Q5 rich	9.0%	6.6%	11.5%	26.4%	27.1%	25.7%	9.2%	6.9%	11.5%	25.4%	27.2%	23.6%	8.9%	6.3%	11.6%	27.5%	27.0%	28.1%
	Absolute	2.8%	1.9%	3.6%	4.7%	5.8%	3.6%	2.0%	1.1%	3.0%	1.6%	2.8%	0.3%	2.7%	1.6%	3.7%	5.9%	5.8%	6.0%
	Relative	1.45	1.40	1.46	1.22	1.27	1.16	1.28	1.19	1.35	1.07	1.11	1.01	1.44	1.34	1.47	1.27	1.27	1.27
Education																			
	Q1 least	6.9%	5.0%	8.9%	25.8%	25.1%	26.6%	n/a	n/a	n/a	n/a	n/a	n/a	6.9%	5.0%	8.9%	25.8%	25.1%	26.6%
	Q2	8.0%	5.3%	10.6%	25.6%	24.8%	26.4%	10.0%	7.6%	12.4%	25.2%	26.6%	23.8%	7.7%	5.1%	10.4%	25.7%	24.6%	26.7%
	Q3	7.8%	5.5%	10.2%	25.8%	25.4%	26.2%	8.5%	6.5%	10.6%	25.5%	27.2%	23.7%	7.7%	5.3%	10.1%	25.9%	25.0%	26.7%
	Q4	8.2%	5.7%	10.6%	24.6%	24.3%	24.8%	9.1%	6.7%	11.5%	23.9%	25.2%	22.6%	7.8%	5.3%	10.3%	24.8%	23.9%	25.7%
	Q5 most	8.5%	5.9%	11.1%	25.1%	25.2%	25.0%	8.5%	6.2%	10.8%	24.9%	26.1%	23.8%	8.5%	5.8%	11.2%	25.2%	24.6%	25.8%
	Absolute	**1.6%**	**0.9%**	**2.2%**	−0.7%	0.1%	**−1.6%**	−1.5%	−1.4%	−1.6%	−0.3%	−0.5%	0.0%	**1.6%**	**0.8%**	**2.3%**	−0.6%	−0.5%	−0.8%
	Relative	**1.23**	**1.18**	**1.25**	0.97	1.00	**0.94**	0.85	0.82	0.87	0.99	0.98	1.00	**1.23**	**1.16**	**1.26**	0.98	0.98	0.97

Note: Q = quintile; Java region includes Bali; Papua region includes Maluku and Nusa Tenggara. Income quintile used district-level poverty rate (e.g., Q1 = 20% of districts with highest poverty rate). Absolute (Relative) = difference (ratio) between Papua and Java as well as Q1 and Q5. For education, Absolute (Relative) was between Q1 and Q5 except among urban areas (Q2 and Q5). Boldface values show statistical significance at 5% level (see Appendix E for the regression outputs).

## Data Availability

Available from the corresponding author upon reasonable request.

## Appendix B. Regression Outputs for Urban/Rural Differences

	**Diagnosed All**	**Diagnosed Males**	**Diagnosed Females**	**Undiagnosed All**	**Undiagnosed Males**	**Undiagnosed Females**
	**Coef**	**Coef**	**Coef**	**Coef**	**Coef**	**Coef**
Rural	Reference					
Urban	1.24 **	1.28 **	1.09 *	−0.80	1.39	−2.97 **
Constant	7.63 **	5.27 **	10.07 **	25.54 **	24.68 **	26.37 **
Observations						
R-squared	513	511	512	514	514	514

Note: Coef = OLS coefficient; significance level ** *p* < 0.01, * *p* < 0.05.

## Appendix C. Ten Districts with LOWEST Prevalence of Diagnosed and Undiagnosed Hypertension among Adults in Indonesia

	**Prevalence**	**Province**	**Region**	**Urban**	**Poverty**	**Education**	**Pop (000)**
(a) Diagnosed all							
Kab. Buton Tengah	0.0%	Central Sulawesi	Sulawesi	Rural	15%	80%	89
Kab. Yahukimo	0.3%	Papua	Papua	Rural	39%	12%	181
Kab. Intan Jaya	0.4%	Papua	Papua	Rural	43%	9%	46
Kab. Dogiyai	0.5%	Papua	Papua	Rural	30%	39%	92
Kab. Pegunungan Bintang	0.6%	Papua	Papua	Rural	31%	21%	72
Kab. Lanny Jaya	0.7%	Papua	Papua	Rural	40%	46%	172
Kab. Mambramo Raya	0.7%	Papua	Papua	Rural	30%	51%	21
Kab. Buru Selatan	1.0%	Maluku	Papua	Rural	16%	67%	59
Kab. Muna Barat	1.2%	Southeast Sulawesi	Sulawesi	Rural	14%	71%	77
Kab. Jayawijaya	1.2%	Papua	Papua	Rural	39%	67%	206
AVERAGE					30%	46%	102
(b) Diagnosed males							
Kab. Yahukimo	0%	Papua	Papua	Rural	39%	12%	181
Kab. Pegunungan Bintang	0%	Papua	Papua	Rural	31%	21%	72
Kab. Tolikara	1%	Papua	Papua	Rural	33%	34%	131
Kab. Dogiyai	1%	Papua	Papua	Rural	30%	39%	92
Kab. Lanny Jaya	1%	Papua	Papua	Rural	40%	46%	172
Kab. Mambramo Raya	1%	Papua	Papua	Rural	30%	51%	21
Kab. Intan Jaya	1%	Papua	Papua	Rural	43%	9%	46
Kab. Jayawijaya	1.1%	Papua	Papua	Rural	39%	67%	206
Kab. Teluk Wondama	1.1%	West Papua	Papua	Rural	33%	39%	30
Kab. Halmahera Barat	1.2%	North Maluku	Papua	Rural	9%	70%	111
AVERAGE					33%	39%	106
(c) Diagnosed females							
Kab. Buton Tengah	0%	Southeast Sulawesi	Sulawesi	Rural	15%	80%	89
Kab. Yahukimo	0%	Papua	Papua	Rural	39%	12%	181
Kab. Dogiyai	0%	Papua	Papua	Rural	30%	39%	92
Kab. Mambramo Raya	0%	Papua	Papua	Rural	30%	51%	21
Kab. Diyai	0.4%	Papua	Papua	Rural	43%	51%	69
Kab. Lanny Jaya	0.6%	Papua	Papua	Rural	40%	46%	172
Kab. Buru Selatan	0.6%	Maluku	Papua	Rural	16%	67%	59
Kab. Muna Barat	0.7%	Southeast Sulawesi	Sulawesi	Rural	14%	71%	77
Kab. Pegunungan Bintang	1.0%	Papua	Papua	Rural	31%	21%	72
Kab. Jayawijaya	1.3%	Papua	Papua	Rural	39%	67%	206
AVERAGE					30%	51%	104
(d) Undiagnosed all							
Kab. Puncak Jaya	7%	Papua	Papua	Rural	36%	21%	115
Kab. Nduga	10%	Papua	Papua	Rural	38%	9%	94
Kab. Tolikara	10%	Papua	Papua	Rural	33%	34%	131
Kab. Asmat	11%	Papua	Papua	Rural	27%	21%	88
Kab. Halmahera Tengah	11%	North Maluku	Papua	Rural	14%	63%	50
Kab. Mimika	12%	Papua	Papua	Rural	15%	67%	201
Kab. Sorong Selatan	12%	West Papua	Papua	Rural	19%	56%	43
Kab. Simeulue	12%	Aceh	Sumatera	Rural	20%	81%	89
Kab. Aceh Jaya	12%	Aceh	Sumatera	Rural	14%	74%	86
Kab. Teluk Wondama	13%	West Papua	Papua	Rural	33%	39%	30
AVERAGE					25%	46%	93
(e) Undiagnosed males							
Kab. Puncak Jaya	6.8%	Papua	Papua	Rural	36%	21%	115
Kab. Halmahera Tengah	9.4%	North Maluku	Papua	Rural	14%	63%	50
Kab. Keerom	10.1%	Papua	Papua	Rural	17%	61%	54
Kab. Aceh Jaya	10.1%	Aceh	Sumatera	Rural	14%	74%	86
Kab. Simeulue	10.5%	Aceh	Sumatera	Rural	20%	81%	89
Kab. Dompu	10.6%	West Nusa Tenggara	Papua	Rural	12%	70%	238
Kab. Nduga	11.2%	Papua	Papua	Rural	38%	9%	94
Kab. Sumbawa	11.6%	West Nusa Tenggara	Papua	Rural	14%	56%	441
Kab. Buton Tengah	11.7%	Southeast Sulawesi	Sulawesi	Rural	15%	80%	89
Kab. Sorong Selatan	11.8%	West Papua	Papua	Rural	19%	56%	43
AVERAGE					20%	57%	130
(f) Undiagnosed females							
Kab. Puncak Jaya	6.2%	Papua	Papua	Rural	36%	21%	115
Kab. Nduga	7.8%	Papua	Papua	Rural	38%	9%	94
Kab. Tolikara	8.4%	Papua	Papua	Rural	33%	34%	131
Kab. Tambrauw	9.6%	West Papua	Papua	Rural	35%	47%	14
Kab. Asmat	10.0%	Papua	Papua	Rural	27%	21%	88
Kab. Mimika	10.5%	Papua	Papua	Rural	15%	67%	201
Kab. Jayawijaya	11.2%	Papua	Papua	Rural	39%	67%	206
Kab. Yahukimo	11.3%	Papua	Papua	Rural	39%	12%	181
Kab. Mambramo Tengah	11.4%	Papua	Papua	Rural	37%	54%	46
Kab. Boven Digul	12.4%	Papua	Papua	Rural	20%	35%	63
AVERAGE					32%	37%	114

Note: Urban = city, Rural = regency; Pop = population. The districts are ordered by prevalence (column 1).

## Appendix D. Ten Districts with HIGHEST Prevalence of Diagnosed and Undiagnosed Hypertension among Adults in Indonesia

	**Prevalence**	**Province**	**Region**	**Urban**	**Poverty**	**Education**	**Pop (000)**
(a) Diagnosed all							
Kab. Sitaro Kepulauan	20.8%	North Sulawesi	Sulawesi	Rural	10%	71%	66
Kota Tomohon	17.7%	North Sulawesi	Sulawesi	Urban	6%	71%	100
Kab. Kep Talaud	16.6%	North Sulawesi	Sulawesi	Rural	10%	71%	89
Kab. Natuna Kep	16.5%	Riau Islands	Sumatera	Rural	5%	70%	74
Kab. Minahasa	16.3%	North Sulawesi	Sulawesi	Rural	7%	65%	329
Kab. Anambas Kep	15.6%	Riau Islands	Sumatera	Rural	7%	77%	40
Kab. Sumedang	15.3%	West Java	Java	Rural	10%	43%	1137
Kab. Tanah Tidung	14.4%	North Kalimantan	Kalimantan	Rural	5%	45%	22
Kab. Minahasa Selatan	14.3%	North Sulawesi	Sulawesi	Rural	9%	62%	205
Kab. Karimun	14.2%	Riau Islands	Sumatera	Rural	7%	70%	225
AVERAGE					8%	65%	229
(b) Diagnosed males							
Kota Tomohon	15.8%	North Sulawesi	Sulawesi	Urban	6%	71%	100
Kab. Puncak Jaya	15.2%	Papua	Papua	Rural	36%	21%	115
Kab. Sitaro Kepulauan	14.2%	North Sulawesi	Sulawesi	Rural	10%	71%	66
Kab. Kep Talaud	13.3%	North Sulawesi	Sulawesi	Rural	10%	71%	89
Kab. Mahakam Ulu	13.2%	East Kalimantan	Kalimantan	Rural	12%	52%	26
Kab. Minahasa	12.9%	North Sulawesi	Sulawesi	Rural	7%	65%	329
Kab. Gianyar	11.5%	Bali	Java	Rural	4%	77%	495
Kab Klungkung	11.2%	Bali	Java	Rural	6%	77%	176
Kab. Tanah Tidung	11.0%	North Kalimantan	Kalimantan	Rural	5%	45%	22
Kab. Natuna Kep	11.0%	Riau Islands	Sumatera	Rural	5%	70%	74
AVERAGE					10%	62%	149
(c) Diagnosed females							
Kab. Sitaro Kepulauan	27.0%	North Sulawesi	Sulawesi	Rural	10%	71%	66
Kab. Anambas Kep	22.4%	Riau Islands	Sumatera	Rural	7%	77%	40
Kab. Natuna Kep	22.4%	Riau Islands	Sumatera	Rural	5%	70%	74
Kab. Sumedang	20.9%	West Java	Java	Rural	10%	43%	1137
Kab. Minahasa Selatan	20.3%	North Sulawesi	Sulawesi	Rural	9%	62%	205
Kab. Minahasa	19.9%	North Sulawesi	Sulawesi	Rural	7%	65%	329
Kab. Kep Talaud	19.7%	North Sulawesi	Sulawesi	Rural	10%	71%	89
Kota Tomohon	19.6%	North Sulawesi	Sulawesi	Urban	6%	71%	100
Kab. Sangihe Kepulauan	19.0%	North Sulawesi	Sulawesi	Rural	12%	55%	130
Kab. Hulu Sungai Utara	19.0%	South Kalimantan	Kalimantan	Rural	6%	55%	225
AVERAGE					8%	64%	239
(d) Undiagnosed all							
Kab. Hulu Sungai Tengah	43.2%	South Kalimantan	Kalimantan	Rural	6%	66%	260
Kab. Tabalong	42.2%	South Kalimantan	Kalimantan	Rural	6%	61%	239
Kab. Mamasa	40.8%	West Sulawesi	Sulawesi	Rural	13%	66%	152
Kab. Ciamis	40.8%	West Java	Java	Rural	7%	51%	1168
Kota Madiun	40.4%	East Java	Java	Urban	4%	80%	175
Kab. Cianjur	40.2%	West Java	Java	Rural	10%	45%	2243
Kab. Barito Kuala	39.1%	South Kalimantan	Kalimantan	Rural	5%	62%	298
Melawi	38.6%	West Kalimantan	Kalimantan	Rural	13%	41%	196
Kab. Karo	38.4%	North Sumatera	Sumatera	Rural	9%	74%	389
Kab. Kutai Barat	38.4%	East Kalimantan	Kalimantan	Rural	9%	60%	146
AVERAGE					8%	61%	527
(e) Undiagnosed males							
Kota Madiun	44.9%	East Java	Java	Urban	4%	80%	175
Kab. Tabalong	43.6%	South Kalimantan	Kalimantan	Rural	6%	61%	239
Kab. Hulu Sungai Tengah	42.5%	South Kalimantan	Kalimantan	Rural	6%	66%	260
Kab. Karo	41.3%	North Sumatera	Sumatera	Rural	9%	74%	389
Kab. Kutai Barat	41.1%	East Kalimantan	Kalimantan	Rural	9%	60%	146
Kota Banjarmasin	40.5%	South Kalimantan	Kalimantan	Urban	4%	55%	675
Kota Singkawang	40.4%	West Kalimantan	Kalimantan	Urban	5%	60%	207
Kab. Buton Selatan	39.6%	Southeast Sulawesi	Sulawesi	Rural	15%	44%	77
Kab. Mamasa	39.2%	West Sulawesi	Sulawesi	Rural	13%	66%	152
Kab. Barito Kuala	39.2%	South Kalimantan	Kalimantan	Rural	5%	62%	298
AVERAGE					8%	63%	262
(f) Undiagnosed females							
Kab. Ciamis	44.6%	West Java	Jawa	Rural	7%	51%	1168
Kab. Hulu Sungai Tengah	43.9%	South Kalimantan	Kalimantan	Rural	6%	66%	260
Melawi	43.0%	West Kalimantan	Kalimantan	Rural	13%	41%	196
Kab. Mamasa	42.4%	West Sulawesi	Sulawesi	Rural	13%	66%	152
Kab. Cianjur	41.9%	West Java	Jawa	Rural	10%	45%	2243
Kab. Tabalong	40.7%	South Kalimantan	Kalimantan	Rural	6%	61%	239
Kab. Indramayu	40.4%	West Java	Jawa	Rural	12%	56%	1691
Kab. Wonosobo	40.4%	Central Java	Jawa	Rural	18%	45%	777
Kab. Nganjuk	40.0%	East Java	Jawa	Rural	12%	63%	1041
Kota Batu	39.8%	East Java	Jawa	Urban	4%	73%	200
AVERAGE					10%	57%	797

Note: Urban = city, Rural = regency; Pop = population. The districts are ordered by prevalence (column 1).

## Appendix E. Regression Outputs for Geographic and Socioeconomic Disparity in Diagnosed and Undiagnosed Hypertension

	**Diagnosed All**	**Diagnosed Males**	**Diagnosed Females**	**Undiagnosed All**	**Undiagnosed Males**	**Undiagnosed Females**
	**Coef**	**Coef**	**Coef**	**Coef**	**Coef**	**Coef**
(a) All districts (N = 514)						
Papua	Reference					
Java	3.05 **	1.60 **	4.29 **	7.72 **	7.07 **	8.45 **
Sumatera	1.88 **	0.74 *	2.95 **	1.32	0.06	2.70 **
Kalimantan	3.65 **	2.23 **	5.13 **	9.36 **	8.77 **	10.00 **
Sulawesi	2.77 **	1.53 **	3.93 **	3.91 **	3.49 **	4.36 **
Income						
Quintile 1 poor	Reference					
Quintile 2	−0.20	−0.41	0.03	2.10 **	1.76 *	2.39 **
Quintile 3	0.15	−0.12	0.37	2.56 **	2.31 **	2.73 **
Quintile 4	0.58	0.18	0.99	1.23	1.47	0.96
Quintile 5 rich	0.64	0.67	0.61	0.21	1.47	−1.06
Education						
Quintile 1 least	Reference					
Quintile 2	0.59	0.09	1.00 *	−0.93	−0.89	−0.90
Quintile 3	0.60	0.35	0.77	−0.27	0.22	−0.72
Quintile 4	0.89 *	0.54	1.16 *	−1.25	−0.62	−1.82 *
Quintile 5 most	1.14 **	0.76 *	1.44 **	−0.65	0.45	−1.73 *
(b) Urban (N = 97)						
Papua	Reference					
Java	3.47 **	2.30 **	4.51 **	7.42 **	7.38 **	7.68 **
Sumatera	1.42	0.84	1.94 *	0.62	−0.09	1.44
Kalimantan	3.99 **	3.11 **	4.87 **	8.44 **	9.12 **	7.81 **
Sulawesi	3.74 **	3.07 **	4.27 **	3.75	4.51	3.21
Income						
Quintile 1 poor	Reference					
Quintile 2	1.15	0.26	2.14	−3.13	−2.96	−3.34
Quintile 3	1.17	0.20	2.21	−1.65	−1.43	−1.95
Quintile 4	−0.46	−1.09	0.24	−4.08	−4.28	−3.99
Quintile 5 rich	0.13	−0.41	0.78	−3.43	−2.69	−4.28
Education						
Quintile 1 least	n/a	n/a	n/a	n/a	n/a	n/a
Quintile 2	Reference					
Quintile 3	−0.79	−0.42	−1.13	0.70	1.38	0.05
Quintile 4	−0.15	−0.13	−0.12	−0.52	−0.08	−0.90
Quintile 5 most	−0.21	−0.23	−0.18	1.96	2.51	1.48
(c) Rural (N = 417)						
Papua	Reference					
Java	3.07 **	1.56 **	4.34 **	7.89 **	7.26 **	8.57 **
Sumatera	2.00 **	0.78 *	3.15 **	1.34	0.16	2.65 **
Kalimantan	3.78 **	2.33 **	5.28 **	9.31 **	9.18 **	9.51 **
Sulawesi	2.74 **	1.39 **	4.00 **	3.92 **	3.48 **	4.38 **
Income						
Quintile 1 poor	Reference					
Quintile 2	−0.28	−0.43	−0.12	2.27 **	1.93 *	2.57 **
Quintile 3	−0.03	−0.24	0.15	2.72 **	2.25 *	3.12 **
Quintile 4	0.66	0.19	1.13	1.81 *	1.76	1.81
Quintile 5 rich	0.38	0.33	0.49	0.71	0.75	0.67
Education						
Quintile 1 least	Reference					
Quintile 2	0.49	−0.03	0.92	−0.88	−0.89	−0.79
Quintile 3	0.61	0.30	0.84	−0.06	0.20	−0.27
Quintile 4	0.72	0.33	1.05	−0.76	−0.52	−0.92
Quintile 5 most	1.15 *	0.74 *	1.54 *	−0.90	−0.14	−1.66

Note: Coef = OLS coefficient; significance level ** *p* < 0.01, * *p* < 0.05.
